# Site-Directed Mutagenesis of VvCYP76F14 (Cytochrome P450) Unveils Its Potential for Selection in Wine Grape Varieties Linked to the Development of Wine Bouquet

**DOI:** 10.1021/acs.jafc.3c09083

**Published:** 2024-02-09

**Authors:** Bin Peng, Jianguo Ran, Yiyang Li, Meiling Tang, Huilin Xiao, Shengpeng Shi, Youzheng Ning, Adeeba Dark, Jin Li, Xueqiang Guan, Zhizhong Song

**Affiliations:** † The Engineering Research Institute of Agriculture and Forestry, Ludong University, Yantai 264025, China; ‡ Cocodala Vocational and Technical College, Cocodala 853213, China; § Jiangsu Vocational College of Agriculture and Forestry, Zhenjiang 212499, China; ∥ Yantai Academy of Agricultural Sciences, Yantai 265599, China; ⊥ Department of Plant Science, University of Cambridge, Cambridge CB2 3EA, U.K.; # Shandong Technology Innovation Center of Wine Grape and Wine/COFCO Great Wall Wine (Penglai) Co., Ltd, Yantai 264000, China

**Keywords:** wine bouquet, cytochrome P450 enzyme, VvCYP76F14, amino acid residue, site-directed mutagenesis, fingerprint marker

## Abstract

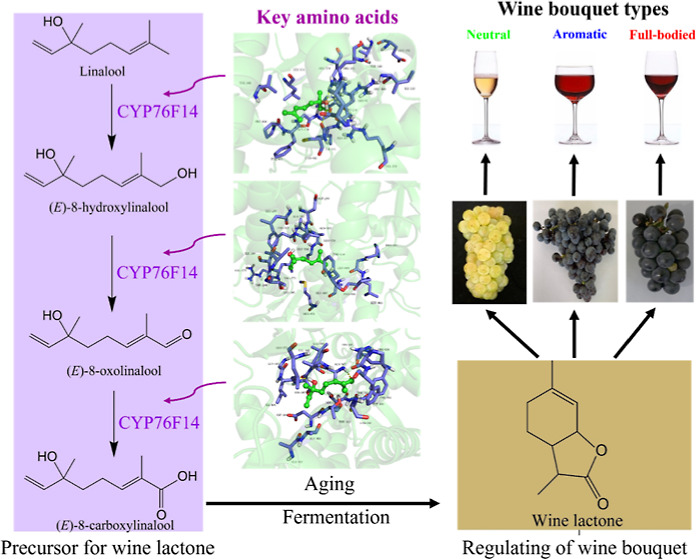

Bouquet is a fascinating wine characteristic that serves as an indicator of wine quality, developing during the aging process. The multifunctional monoterpenol oxidase VvCYP76F14 in wine grapes sequentially catalyzes three reactions to produce (*E*)-8-carboxylinalool, a crucial precursor for wine bouquet. Previous studies indicated that the activity of VvCYP76F14 derived from different wine grape varieties did not correlate with the amino acid sequence differences. In this study, 54 wine grape varieties were categorized into neutral, aromatic, and full-bodied types based on the sequence differences of VvCYP76F14, closely correlated with the content of wine lactone precursors. Computer modeling and molecular docking analysis of the full-bodied CYP76F14 revealed 17, 19, and 18 amino acid residues in the VvCYP76F14–linalool, VvCYP76F14–(*E*)-8-hydroxylinalool, and VvCYP76F14–(*E*)-8-oxolinalool complexes, respectively. Site-directed mutagenesis and in vitro enzyme activity analysis confirmed the substitutions of the key amino acid residues in neutral and aromatic varieties. Notably, the D299 mutation of VvCYP76F14 resulted in the complete loss of (*E*)-8-oxolinalool and (*E*)-8-carboxylinalool activities, aligning with the undetectable levels of (*E*)-8-oxolinalool and (*E*)-8-carboxylinalool in “Yantai 2-3-37”, which harbors the D299T substitution. Favorably, VvCYP76F14 could serve as a cost-effective fingerprint marker for screening superior hybrid offspring with the desired levels of wine lactone precursors.

## Introduction

Aroma plays a crucial role as a sensory indicator of wine quality and remains a highly captivating aspect of wine consumption.^1−4^ The aroma of wine primarily originates from two sources. One source is the flavor compounds present in the berries of wine grape (*Vitis vinifera* L.), giving rise to sensory characteristics, such as floral, sweet, herbal, and fruity notes. These aromas are referred to as primary aromas.^1,5^ The other source of wine aroma is the formation of new compounds, known as wine bouquets or secondary aromas. These are generated through biochemical processes during fermentation and aging, where flavor precursors undergo transformations to produce a wide range of aromatic compounds.^4,6,7^ During the aging process of wine, the primary aromas derived from the wine grape tend to diminish over time, while the wine bouquet becomes more pronounced, imparting the typical aroma characteristics commonly associated with the wine. As the wine matures and develops through aging, the evolving bouquet plays a significant role in defining its unique aromatic profile.^3,8,9^

In recent decades, the application of modern analytical techniques, in combination with sensory evaluation methods, has unveiled a vast array of compounds contributing to the complex and diverse wine bouquet.^1,10,11^ Notably, only a relatively restricted number of compounds, including sesquiterpenes, fusel alcohols, esters, and lactones, have been recognized as playing a pivotal role in shaping the typical wine bouquet.^1^

**Figure 1:**
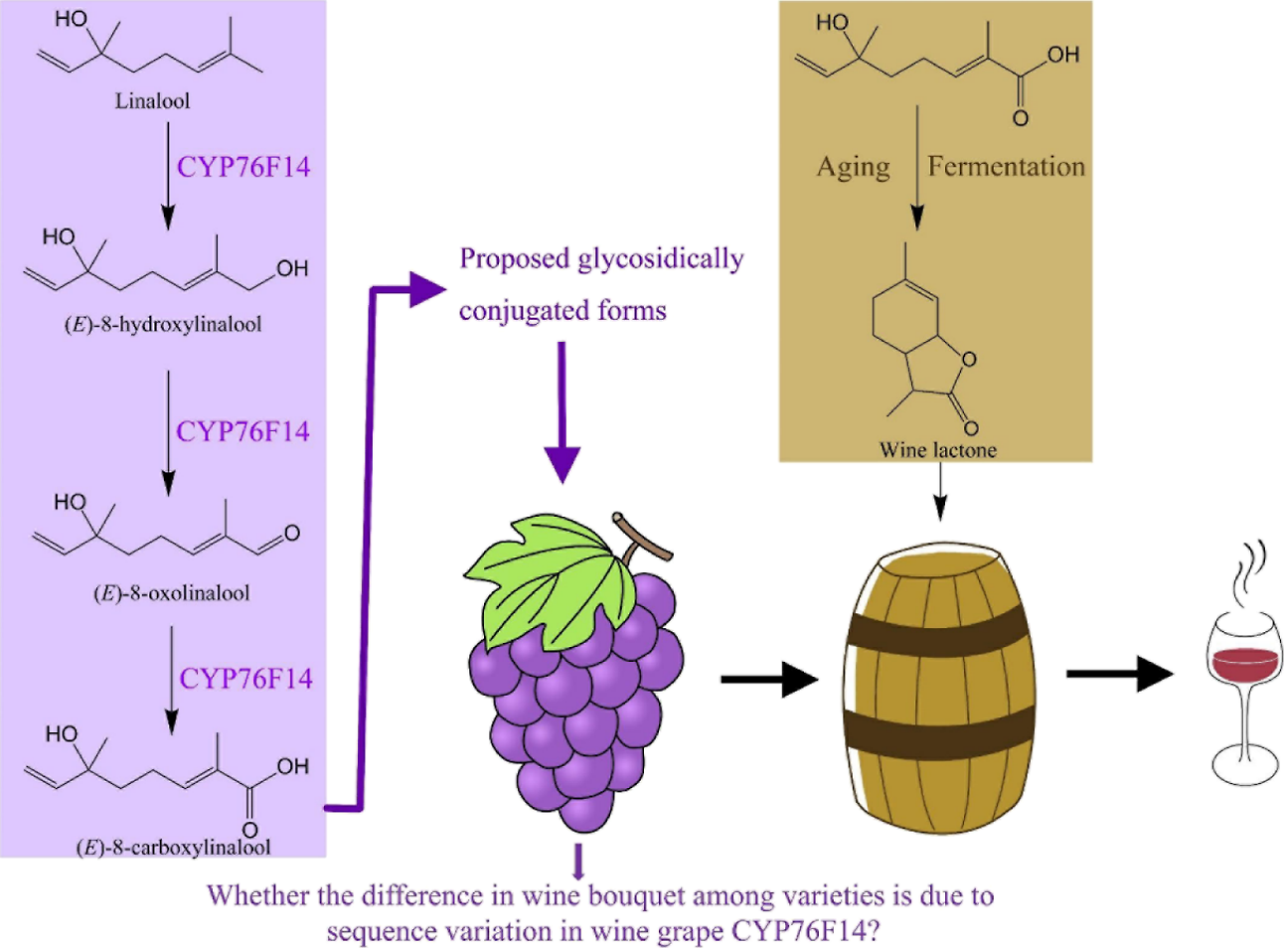
Putative pathway of wine lactone and its precursors’ biosynthesis.

Previous studies have emphasized the crucial role of bicyclic monoterpene lactones in regulating wine bouquet due to their pleasant odor and remarkably low odor threshold. It is important to note that bicyclic monoterpene lactones are not formed in the grape berry itself but are generated during wine aging, derived from the crucial precursor (*E*)-8-carboxylinalool (Figure 1).^12,13^ In particular, the biosynthesis of (*E*)-8-carboxylinalool in grape berries involves a three-step enzymatic process catalyzed by the enzyme VvCYP76F14, a cytochrome P450 enzyme found in grapevine. Initially, VvCYP76F14 catalyzes the hydroxylation of linalool at C8, resulting in the formation of (*E*)-8-hydroxylinalool. Subsequently, (*E*)-8-hydroxylinalool undergoes dehydrogenation oxidation at the same carbon atom, leading to the production of (*E*)-8-oxolinalool. Finally, (*E*)-8-oxolinalool is transformed into (*E*)-8-carboxylinalool through oxidation (Figure 1).^11,13−15^ In contrast to the majority of plant monofunctional P450s that catalyze a single reaction, the CYP76 family is characterized by its ability to function as a multifunctional monooxygenase that can catalyze multiple reactions.^16^ In *Arabidopsis thaliana*, AtCYP76C1 can catalyze the conversion of linalool into several compounds, including 8-hydroxylinalool, 8-oxolinalool, and 8-carboxylinalool, as well as the formation of lilac aldehydes and lilac alcohols.^17,18^ Notably, VvCYP76 belongs to the class II monooxygenase P450 system, and the redox partner NADPH cytochrome P450 reductase (CPR) acts as the electron donor, transferring electrons from NADPH to the P450 enzyme, enabling its catalytic activity.^11,19−22^ The highly conserved class II monooxygenase region of the CYP450s features the general active motif signature A(G)G(A)XD(E)T.^23−25^ However, the key amino acid residues responsible for each catalytic step in the three reaction processes involved in the conversion of linalool to (*E*)-8-carboxylinalool remain unknown. Previous studies demonstrated differences in amino acid sequences between CYP76F14s derived from two wine grape varieties, with no significant differences in enzyme activity in vitro.^13^ Whether the difference in the wine bouquet among varieties is due to sequence variation in wine grape CYP76F14 also remains unclear.

According to the studies of Picard et al.,^26^ there is a limited correlation between wine bouquet and the berry aroma derived directly from wine grape berries. Indeed, selecting wine grape varieties solely based on the berry-derived aroma for wine bouquet improvement can be challenging. Previous studies have highlighted numerous genetic differences among hybrid varieties that have the potential to enhance wine bouquet quality.^11,27,28^ However, relying solely on chemical contents to determine the bouquet potential is hindered by variations in grape berries caused by different cultivation environments.^29^ Therefore, there is a need to develop rapid and reliable techniques that can aid in the selection of wine grape varieties capable of producing the desired amounts of lactone precursors.

The National Grape Germplasm Repository in Yantai, China, has played a significant role in conserving and maintaining a diverse collection of grape varieties, including those from the *V. vinifera* species and interspecific crosses involving non-vinifera species. The core collection has served as a valuable resource for studying and evaluating the contributions of various wine grape varieties to wine bouquet.^30,31^ According to the National Standards of the People’s Republic of China (GB/T 15037-2006, January 2022) and previous studies on wine,^1,3,12,31−33^ “neutral” (low bouquet density), “aromatic” (middle bouquet density), and “full-bodied” (high bouquet density) are the standard descriptions of grape variety contributing to the wine bouquet. Notably, wine grape VvCYP76F14 may possess catalytic functionality toward multiple substrates, including linalool, (*E*)-8-hydroxylinalool, (*E*)-8-oxolinalool, and (*E*)-8-carboxylinalool, which are important wine lactone precursors.^1,13^ In contrast to previous findings,^26^ this study initially reveals substantial variations in the catalytic activity of VvCYP76F14 among 54 wine grape varieties or superior lines. These differences in activity can be attributed to sequence variations among VvCYP76F14s, ultimately influencing their distinct wine bouquet intensities. Subsequently, we employed computer modeling, molecular docking, and site-directed mutagenesis to identify the key amino acid residues responsible for each catalytic step in the three reaction processes. This study provides a solid foundation for future investigations aimed at exploring the potential application of VvCYP76F14 as a fingerprint marker for selecting wine grape varieties or superior lines capable of producing the desired amounts of wine lactone precursors.

## Materials and Methods

### Chemicals

**Figure 2:**
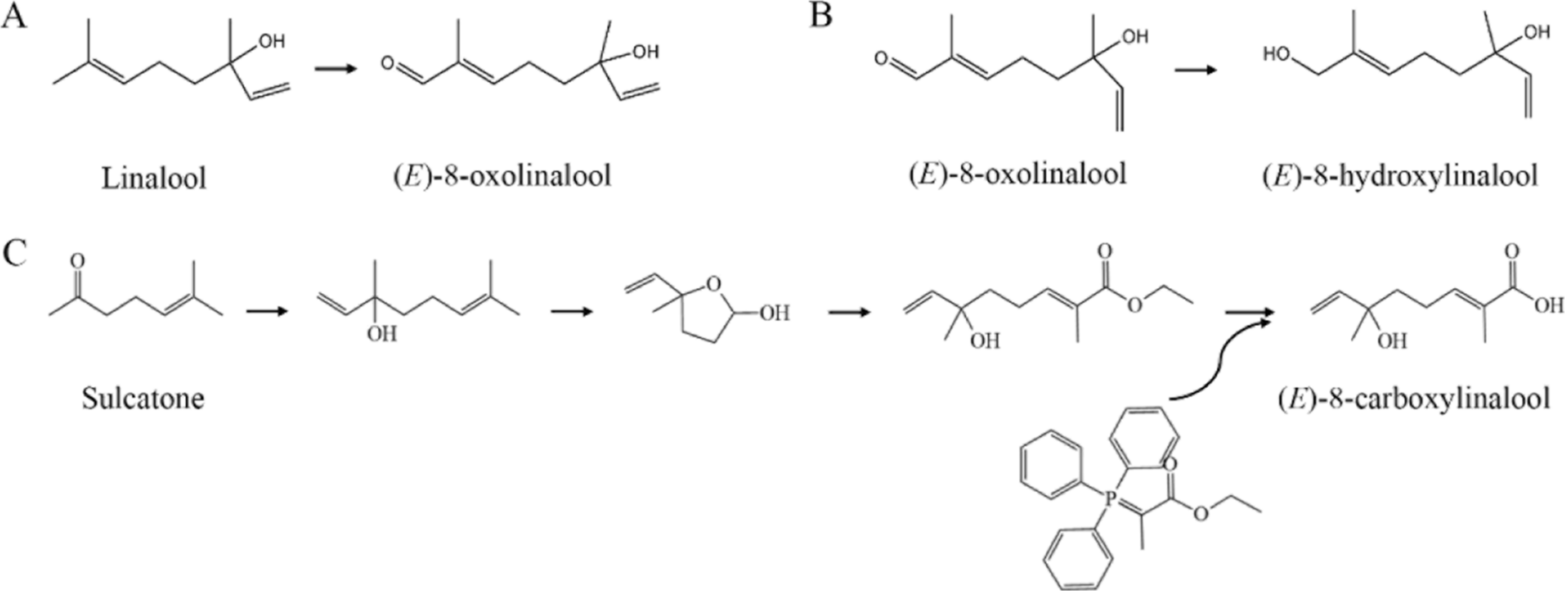
Synthesis pathways of (*E*)-8-oxolinalool (A), (*E*)-8-hydroxylinalool (B), and (*E*)-8-carboxylinalool (C).

The chemicals of (*E*)-8-hydroxylinalool, (*E*)-8-oxolinalool, and (*E*)-8-carboxylinalool were synthesized and purified in their forms by Accela ChemBio Co., Ltd. (Shanghai, China) (Figure 2). The key precursor of linalool was purchased from J and K Scientific Co., Ltd. (Shanghai, China).

### Wine Grape Varieties and Wine Samples

According to the description of the Grape Grower’s Handbook,^34^ berries of 54 wine grape varieties or superior lines were collected from the National Grape Germplasm Repository in Yantai, China, at similar 80% ripening levels, respectively. Three biological replicates were performed, each with 20 individual berries.

All of the wine samples used in this study were generously provided by the Shandong Technology Innovation Center of Wine Grape and Wine/COFCO Great Wall Wine (Penglai) Co., Ltd., located in Yantai, China. These samples were collected from the same vineyard, and the winemaking processes, including fermentation and maturation, adhered to the same established standards,^33,35^ guaranteeing uniformity across the wine production.

### Correlation between Wine Bouquet and Linalool-Derived Compounds in Berry

Sensory evaluation analyses were conducted in a controlled sensory analysis chamber following the guidelines outlined in GB/T 15037-2006 (January 2022). Visual characteristics (color, clarity, and any sediment present), aromatic attributes (fruity, floral, herbal, and any other specific scents), and taste profiles (sweetness, acidity, bitterness, and other flavor components) of grape wine were evaluated by a panel of 20 individuals. The panel underwent uniform training at the Shandong Technology Innovation Center of Wine Grape and Wine/COFCO Great Wall Wine (Penglai) Co., Ltd. The content of linalool, (*E*)-8-hydroxylinalool, (*E*)-8-oxolinalool, and (*E*)-8-carboxylinalool in the wine grape berries was determined using ultraperformance liquid chromatography–mass spectrometry (UPLC–MS) (Waters, Milford, MA, USA), as previously described.^13^ It should be noted that some of the (*E*)-8-carboxylinalool and linalool derivatives present in the berries were glycosylated. To account for these compounds, acid hydrolysis of the samples was conducted at pH 3, allowing for the complete characterization of these compounds.^36^ The principal coordinate analysis (PCA) was performed using Origin 2022 online software (www.originlab.com) to analyze and visualize the data obtained from the sensory evaluation and chemical analysis.

### Isolation and Sequence Analysis of VvCYP76F14

To determine whether the variations in aroma among wine grape varieties were linked to sequence differences, the coding sequences (CDSs) of VvCYP76F14 were cloned from 54 wine grape varieties or superior lines. Total RNA was extracted from the grape samples using a MiniBEST Plant RNA extraction kit (TaKaRa, Dalian, China), and any remaining DNA contamination was removed using the RNase-free recombinant DNase I (TaKaRa, Dalian, China). The quantity and quality of the extracted RNA were assessed using an Invitrogen Qubit Flex Fluorometer (Thermo Fisher Scientific, Waltham, USA). The first-strand cDNA was synthesized by using a PrimeScript II First Strand cDNA Synthesis Kit (TaKaRa, Dalian, China).

The CDSs of VvCYP76F14 were amplified using the PrimeSTAR HS DNA polymerase (TaKaRa, Dalian, China) with the specific primer pairs (forward: 5′-ATGGAGTTGTTGAGTTGTCTG-3′; reverse: 5′-TCAAACCCGTACAGGTAGAGCTTGCAG-3′) as described by Ilc et al.^13^ The PCR products were then cloned into pMD 18-T (TaKaRa, Dalian, China) and sent for sequencing by Shenggong Bioengineering Co., Ltd. (Shanghai, China). The resulting sequences were analyzed and translated into amino acid sequences using the DNAMAN software. A multiple alignment analysis of wine grape VvCYP76F14 proteins was performed using the ClustalW program within the MEGA 13.0 software. A phylogenetic tree of VvCYP76F14 proteins from 54 wine grape varieties or superior lines was constructed using the maximum-likelihood method in MEGA 13.0, based on the amino acid sequence variations.

### Real-Time Quantitative PCR

The qPCR analysis was conducted using a LightCycler 480 system (Roche, Inc., Basel, Switzerland) and SYBR Green qPCR Master Mix (TaKaRa, Dalian, China). Absolute quantification was performed using a standard curve. The positive recombinant VvCYP76F14-PMD 18-T plasmids mentioned above were extracted using a plasmid miniprep kit (Tiangen, Beijing, China) according to the manufacturer’s instructions. The concentration of the extracted plasmids was determined using a Qubit Flex Fluorometer (Thermo Fisher Scientific, Waltham, USA), and copy numbers for the individual genes were calculated. To generate a standard curve, each recombinant plasmid was diluted through eight gradients (10° to 10^–7^) and used as a template for qPCR. The primer pair used for qPCR was as follows: F: 5′-TGTTATCCAACACCATAT-3′; R: 5′-TCCCAGCTTCCTCCATCACA-3′. The first-strand cDNA was synthesized using a PrimeScript II 1st strand cDNA synthesis kit (TaKaRa, Dalian, China) following the manufacturer’s instructions. All cDNA samples were diluted 10 times with RNase-free water before being used as a template for qPCR and subsequent analyses.

### Molecular Docking Analysis

MODELLER v9.19 (http://salilab.org/modeller/) was utilized for homology modeling of the VvCYP76F14 protein from “L35” (full-bodied), using the crystal structure of a *Salvia miltiorrhiza* CYP 450 protein (Protein Data Bank no. 5YLW) as the template.^37^ The protein underwent an energy minimization treatment and served as the receptor structure for molecular docking. The stereochemical quality of the 3D model of VvCYP76F14 was evaluated using PROCHECK and Verify3D.^37,38^ The structures of the substrates (linalool, (*E*)-8-hydroxylinalool, and (*E*)-8-oxolinalool) were optimized using the MOPAC program and constructed using AutoDock4.2.^39^ The Amber14 force field was utilized to perform energy optimization, ensuring the exclusion of any unreasonable spatial structures and stabilization of the binding models.^40^ Molecular dynamics simulations were carried out to estimate the relative binding free energy (Δ*G*_bind_) of the VvCYP76F14-ligand complexes, using the molecular mechanics–generalized Born surface area method.^38^

### Site-Directed Mutagenesis and Heterologous Expression of VvCYP76F14 in *Escherichia coli*

To investigate the catalytic activities of key amino acid residues in VvCYP76F14 from “L35”, the alanine-scanning method was employed to generate alanine-substituted VvCYP76F14-SMs.^38^ To improve the production and facilitate the folding of recombinant VvCYP76F14 proteins, the pMAL-c6T vector (New England Biolabs, Beijing, China) containing a maltose-binding protein (MBP) tag was used for heterologous expression.^41^ The complete CDSs of the VvCYP76F14s and VvCYP76F14-SMs were triplet-code-optimized and synthesized by GenScript Co., Ltd. (Nanjing, China). The correctness of the pMAL-c6T-VvCYP76F14 constructs was confirmed by sequencing (Biomarker Co., Ltd., Beijing, China). These confirmed constructs were then expressed in the *E. coli* BL21 strain (TaKaRa, Dalian, China). The MBP-VvCYP76F14s were further purified using the NEBExpress MBP Fusion and Purification System (New England Biolabs, Hitchin, UK) following the manufacturer’s description.

### In Vitro Enzymatic Activity Assay

In vitro enzyme activity levels of recombinant VvCYP76F14 from “L35” (full-bodied) and VvCYP76F14-SMs were independently assayed using linalool, (*E*)-8-hydroxylinalool, and (*E*)-8-oxolinalool as the substrate. To reconstitute the membrane-bound monooxygenase system and minimize the interference from multiple NADPH CPR homologues present in wine grape itself, the *Arabidopsis* CPR (ATR1)^18,19^ was selected as the electron transport redox partner of VvCYP76F14. The VvCYP76F14 enzyme assays were performed in a total reaction volume of 5 mL with 100 mM Na^+^/K^+^ phosphate buffer (pH 5.0), with varying substrate concentrations, 1 mM NADPH, and adjusted enzyme amounts (VvCYP76F14/ATR1 ≈ 2:1). The reactions were carried out at 26 °C for 1 h with agitation, and the resulting product was collected and analyzed using UPLC–MS (Waters, Milford, MA, USA).^13^ Boiled protein (nonfunctional) was used as a control. All assays were performed in sextuplicate. For the determination of kinetic parameters, substrate reduction was qualitatively and quantitatively determined by UPLC–MS/MS. The turnover number (*k*_cat_) and affinity (*k*_m_) were calculated using Origin 2018 online software (www.originlab.com).

## Results

### Significant Variations in Wine Lactone Precursors among Distinct Wine Grape Berries

The levels of wine lactone precursors (linalool, (*E*)-8-hydroxylinalool, (*E*)-8-oxolinalool, and (*E*)-8-carboxylinalool) were analyzed in the berries of the 54 varieties or superior lines using UPLC–MS. No significant differences in the linalool content were observed among varieties with different bouquet types (Table 1). However, significant variations were observed in the levels of (*E*)-8-hydroxylinalool, (*E*)-8-oxolinalool, and (*E*)-8-carboxylinalool (Table 1). Notably, undetectable levels of (*E*)-8-oxolinalool and (*E*)-8-carboxylinalool production were observed in *V. vinifera* cv. “Yantai 2-3-37” (Table 1). Corresponding wine bouquet evaluations demonstrate that 54 varieties or superior lines were categorized as neutral, aromatic, and full-bodied types, according to the description of Goldammer,^34^ over a span of 5 successive years (Table 1).

**Table 1:** Precursor Content Analysis of Linalool, (*E*)-8-Hydroxylinalool, (*E*)-8-Oxolinalool, and (*E*)-8-Carboxylinalool from 54 Wine Grape Varieties or Superior Lines^a^

wine grape	linalool (μg·g^–1^ FW)	(*E*)-8-hydroxylinalool (μg·g^–1^ FW)	(*E*)-8-oxolinalool (μg·g^–1^ FW)	(*E*)-8-carboxylinalool (μg·g^–1^ FW)	corresponding wine bouquet evaluation
V. vinifera cv. Yantai 1-2-13	4.9228 ± 0.6227^a^	0.2049 ± 0.0195^c^	0.1606 ± 0.0178^c^	0.0597 ± 0.0070^c^	neutral
V. vinifera cv. Yantai 2-1-2	5.9208 ± 0.4611^a^	0.2611 ± 0.0249^c^	0.1953 ± 0.0200^c^	0.0821 ± 0.0051^c^	neutral
V. vinifera cv. Yantai 2-1-7	5.2899 ± 0.5981^a^	0.1839 ± 0.0174^c^	0.1572 ± 0.1711^c^	0.0687 ± 0.0092^c^	neutral
V. vinifera cv. Yantai 2-1-10	4.6601 ± 0.6432^a^	0.2282 ± 0.0199^c^	0.1720 ± 0.0106^c^	0.0601 ± 0.0055^c^	neutral
V. vinifera cv. Yantai 2-2-43	5.1712 ± 0.5521^a^	0.1896 ± 0.0201^c^	0.1589 ± 0.1448^c^	0.0814 ± 0.0073^c^	neutral
V. vinifera cv. Cabernet Franc	4.9123 ± 0.4328^a^	0.2159 ± 0.0257^c^	0.1791 ± 0.0201^c^	0.0797 ± 0.0041^c^	neutral
V. vinifera cv. Sylvanske Cervene	4.7923 ± 0.5279^a^	0.2491 ± 0.0338^c^	0.1484 ± 0.0118^c^	0.0710 ± 0.0090^c^	neutral
V. vinifera cv. Ugni Blanc	5.0567 ± 0.5667^a^	0.1900 ± 0.0144^c^	0.1968 ± 0.0143^c^	0.0645 ± 0.0049^c^	neutral
V. vinifera cv. Mandovank	4.7918 ± 0.5398^a^	0.1947 ± 0.0133^c^	0.1740 ± 0.0206^c^	0.0598 ± 0.0073^c^	neutral
V. vinifera cv. Grenache Noir	4.6093 ± 0.5221^a^	0.2320 ± 0.0355^c^	0.1966 ± 0.0178^c^	0.0627 ± 0.0088^c^	neutral
V. vinifera cv. Malvasia	4.7721 ± 0.5978^a^	0.1825 ± 0.0221^c^	0.1618 ± 0.0203^c^	0.0780 ± 0.0062^c^	neutral
V. vinifera cv. Bacator Red	4.6207 ± 0.6628^a^	0.2299 ± 0.0322^c^	0.1735 ± 0.0158^c^	0.0889 ± 0.0083^c^	neutral
V. vinifera×V. amurensis cv. Gongniang No. 1	4.8303 ± 0.6128^a^	0.1999 ± 0.0213^c^	0.1620 ± 0.0188^c^	0.0828 ± 0.0062^c^	neutral
V. vinifera cv. GF-L43-31	4.9773 ± 0.6478^a^	0.2053 ± 0.0185^c^	0.1665 ± 0.0179^c^	0.0778 ± 0.0047^c^	neutral
V. vinifera cv. Ewe Tail	6.0436 ± 0.4412^a^	0.2365 ± 0.0354^c^	0.1948 ± 0.0257^c^	0.0736 ± 0.0083^c^	neutral
V. vinifera cv. Italian Riesling	5.9578 ± 0.6621^a^	0.1504 ± 0.0201^c^	0.1881 ± 0.0224^c^	0.0833 ± 0.0091^c^	neutral
V. vinifera cv. GF-L41-44	5.0084 ± 0.8871^a^	0.1971 ± 0.0214^c^	0.1629 ± 0.0147^c^	0.0815 ± 0.0086^c^	neutral
V. vinifera cv. Syrah5BB	4.8389 ± 0.6255^a^	0.2393 ± 0.0324^b^	0.1984 ± 0.0212^c^	0.0854 ± 0.0077^c^	neutral
V. vinifera cv. GF-B2-11	4.9578 ± 0.6621^a^	0.1504 ± 0.0201^c^	0.1881 ± 0.0224^c^	0.0636 ± 0.0072^c^	neutral
V. vinifera×V. amurensis cv. Gongniang No. 2	5.2384 ± 0.8871^a^	0.1971 ± 0.0214^c^	0.1629 ± 0.0147^c^	0.0629 ± 0.0081^c^	neutral
V. vinifera cv. Chardonnay	6.0321 ± 0.4186^a^	0.1791 ± 0.0236^c^	0.1822 ± 0.0314^c^	0.0894 ± 0.0079^c^	neutral
V. vinifera cv. Dunkelfelder	4.6879 ± 0.5162^a^	0.2107 ± 0.0242^c^	0.1793 ± 0.0213^c^	0.0705 ± 0.0084^c^	neutral
V. vinifera cv. Yantai 2-3-37	4.9033 ± 0.5522^a^	0.1325 ± 0.0141^c^	N.D.	N.D.	neutral
V. vinifera cv. Cabernet Sauvignon	4.6371 ± 0.5758^a^	0.2359 ± 0.0211^c^	0.2031 ± 0.0246^c^	0.0813 ± 0.0079^c^	neutral
V. vinifera cv. Pinot Gris	4.5481 ± 0.7265^a^	1.0176 ± 0.0964^b^	0.7502 ± 0.0813^b^	0.3169 ± 0.0301^b^	aromatic
V. vinifera cv. Hedavas	4.6469 ± 0.8371^a^	0.8015 ± 0.0821^b^	0.7628 ± 0.0910^b^	0.2863 ± 0.0177^b^	aromatic
V. vinifera cv. Slankamenka Biele	5.9745 ± 0.7154^a^	1.1522 ± 0.1557^b^	0.6849 ± 0.0580^b^	0.2993 ± 0.0300^b^	aromatic
V. vinifera cv. GF-67-198-3	4.7796 ± 0.6741^a^	1.2182 ± 0.0982^b^	0.8387 ± 0.0901^b^	0.2860 ± 0.0211^b^	aromatic
V. vinifera cv. GF-48-12	4.8085 ± 0.6517^a^	0.9924 ± 0.0841^b^	0.8552 ± 0.0612^b^	0.3467 ± 0.0199^b^	aromatic
V. vinifera×V. amurensis cv. Beichun	5.5698 ± 0.7469^a^	1.0739 ± 0.1441^b^	0.7862 ± 0.0599^b^	0.3955 ± 0.0403^b^	aromatic
V. vinifera cv. Viognier	5.8281 ± 0.3641^a^	1.2720 ± 0.1583^b^	0.8224 ± 0.0701^b^	0.3199 ± 0.0297^b^	aromatic
V. vinifera cv. Marselan	5.2877 ± 0.6258^a^	1.3653 ± 0.0944^b^	0.8967 ± 0.0359^b^	0.3444 ± 0.0433^b^	aromatic
V. vinifera cv. Muscat Ottonel	4.9802 ± 0.6968^a^	1.1850 ± 0.1541^b^	0.9038 ± 0.0813^b^	0.3304 ± 0.0274^b^	aromatic
V. vinifera×V. amurensis cv. Yanniang No. 1	6.3756 ± 0.4872^a^	1.2038 ± 0.1821^b^	0.7972 ± 0.0910^b^	0.2873 ± 0.0199^b^	aromatic
V. vinifera×V. amurensis cv. Yanniang No. 4	5.4916 ± 0.3741^a^	1.3273 ± 0.1997^b^	0.8500 ± 0.0447^b^	0.2618 ± 0.0246^b^	aromatic
V. vinifera cv. Merlot	5.2779 ± 0.7872^a^	0.9187 ± 0.0458^b^	0.7045 ± 0.0299^b^	0.3351 ± 0.0410^b^	aromatic
V. vinifera cv. Cabernet Gernischt	5.1508 ± 0.7644^a^	0.8899 ± 0.0911^b^	0.7715 ± 0.0618^b^	0.3209 ± 0.0241^b^	aromatic
V. vinifera cv. Yan73	5.8784 ± 0.3661^a^	0.9882 ± 0.1001^b^	0.7474 ± 0.0410^b^	0.2654 ± 0.0143^b^	aromatic
V. vinifera cv. Zhiliranse	5.4182 ± 0.7485^a^	1.3239 ± 0.2008^b^	1.0010 ± 0.1820^b^	0.3069 ± 0.0192^b^	aromatic
V. vinifera cv. Yantai 1-2-8	5.7930 ± 0.6278^a^	0.9403 ± 0.0874^b^	1.0109 ± 0.0987^b^	0.2915 ± 0.0311^b^	aromatic
V. vinifera cv. Yantai 1-2-9	4.9323 ± 0.6347^a^	1.4185 ± 0.1084^b^	0.9320 ± 0.0610^b^	0.4226 ± 0.0586^b^	aromatic
V. vinifera cv. Yantai 2-2-2	5.5042 ± 0.6287^b^	0.9172 ± 0.0921^b^	0.8602 ± 0.0778^b^	0.3008 ± 0.0447^b^	aromatic
V. vinifera cv. Yantai 2-1-8	5.2625 ± 0.5478^a^	1.2230 ± 0.1679^b^	0.8550 ± 0.0952^b^	0.2763 ± 0.0401^b^	aromatic
V. vinifera cv. Yantai 2-1-3	5.4296 ± 0.6477^a^	0.9880 ± 0.0558^b^	0.7901 ± 0.0654^b^	0.3124 ± 0.0196^b^	aromatic
V. vinifera cv. Yantai 2-1-1	5.5593 ± 0.3117^a^	0.8603 ± 0.0927^b^	0.8584 ± 0.0799^b^	0.2913 ± 0.0411^b^	aromatic
V. vinifera×V. labrusca cv. Heihuxiang	5.7924 ± 0.4859^a^	3.3439 ± 0.5117^a^	2.3190 ± 0.1447^a^	0.8513 ± 0.1003^a^	full-bodied
V. vinifera×V. labrusca cv. Meizhouzi	6.4987 ± 0.8364^a^	3.3135 ± 0.2998^a^	1.8515 ± 0.1558^a^	0.9170 ± 0.0844^a^	full-bodied
V. vinifera×V. labrusca cv. Concord	6.0451 ± 0.5227^a^	3.4265 ± 0.3554^a^	1.9614 ± 0.2177^a^	0.8608 ± 0.0677^a^	full-bodied
V. vinifera×V. labrusca cv. Yanniang No. 2	6.1150 ± 0.7193^a^	3.9729 ± 0.2241^a^	2.3293 ± 0.0975^a^	0.9507 ± 0.0974^a^	full-bodied
V. vinifera×V. labrusca cv. Yanniang No. 3	4.2539 ± 0.7871^a^	2.9753 ± 0.3109^a^	1.8608 ± 0.2104^a^	0.8889 ± 0.0894^a^	full-bodied
V. vinifera×V. labrusca cv. L35	6.6051 ± 0.9551^a^	2.7829 ± 0.3500^a^	1.8105 ± 0.2887^a^	0.8864 ± 0.0716^a^	full-bodied
V. vinifera×V. labrusca cv. Hesse Bier	5.5292 ± 0.4379^a^	4.0809 ± 0.3870^a^	1.6172 ± 0.1994^a^	0.7821 ± 0.0912^a^	full-bodied
V. vinifera×V. labrusca cv. L42	6.0073 ± 0.4007^a^	3.5031 ± 0.4112^a^	1.9291 ± 0.0744^a^	0.9060 ± 0.1108^a^	full-bodied
V. vinifera×V. labrusca cv. Yantai 2-2-1	6.0501 ± 0.8726^a^	2.9794 ± 0.1993^a^	1.7934 ± 0.2098^a^	0.9162 ± 0.1172^a^	full-bodied

a Data were presented as the mean ± SE (*n* = 3). Letters represent significant differences at *P* ≤ 0.05, as determined using ANOVA followed by Fisher’s LSD test. N.D., not detectable.

### Amino Acid Residue Mutations Identified in VvCYP76F14s from Neutral and Aromatic Wine Grape Varieties

**Figure 3:**
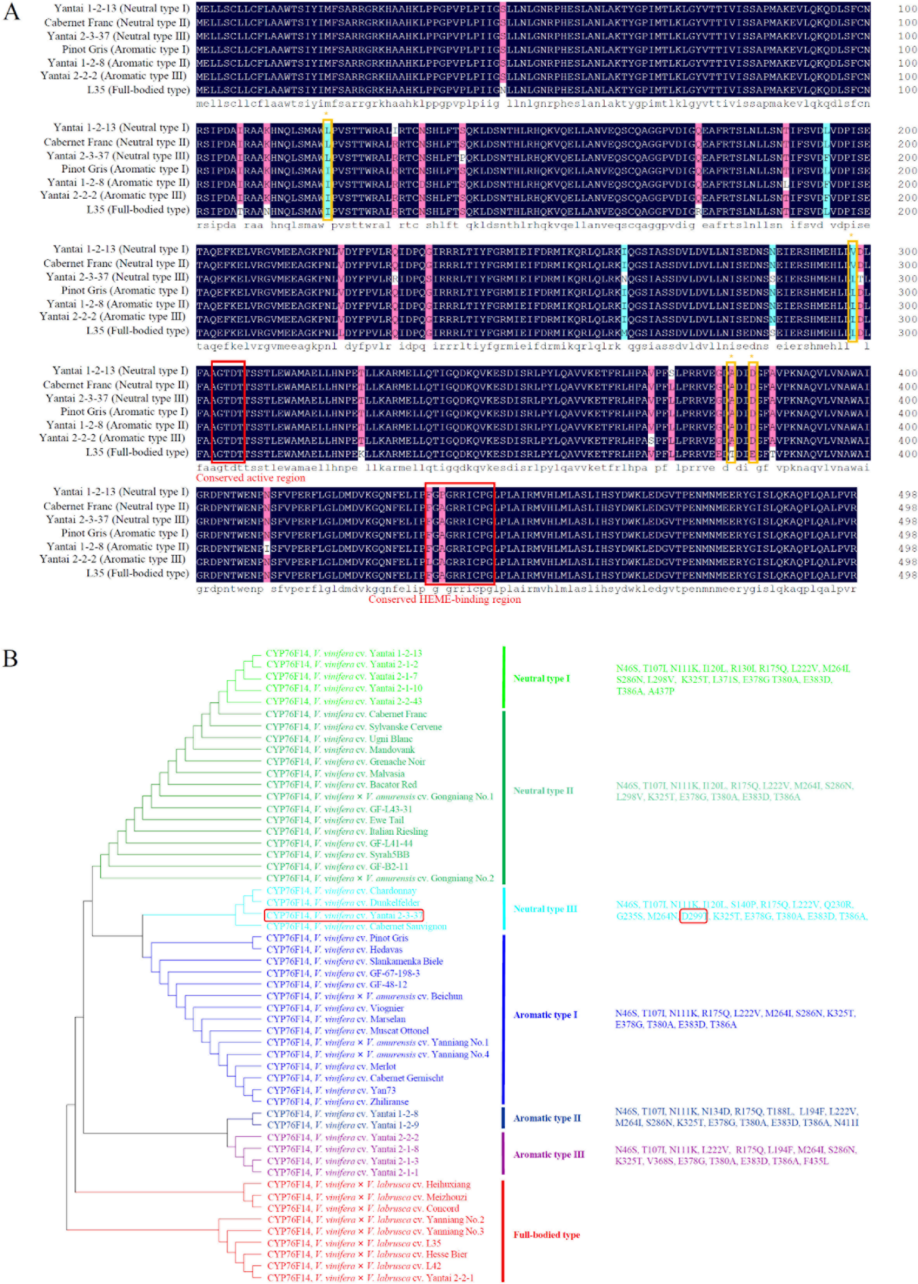
Amino acid sequence alignment and phylogenetic tree construction of VvCYP76F14s from 54 wine grape varieties or superior lines. (A) Amino acid sequence alignment revealed amino acid residue mutations in VvCYP76F14s derived from neutral- and aromatic-type berries. Multiple sequence alignment was performed on the amino acid sequences of VvCYP76F14s from 7 wine grape varieties or superior lines representing neutral, aromatic, and full-bodied types, respectively. The alignment analysis was conducted using the ClustalW program within the MEGA 13.0 software. Mutated amino acids in the neutral variety were indicated by brown frames, and in the aromatic variety, acids were indicated by stars. Conserved regions are highlighted in red frames. (B) Phylogenetic analysis revealed key amino acid residue mutations in VvCYP76F14s. The phylogenetic tree was constructed using VvCYP76F14 protein sequences from 54 wine grape varieties or superior lines, employing the maximum-likelihood method in the MEGA 13.0 software.

To further validate the relationship between amino acid substitution and wine bouquet intensity, the CDSs of VvCYP76F14s were isolated from 54 wine grape varieties or superior lines (Figure 3). Sequencing results showed that sequence variations were identified among these three types of wine grape varieties (Figure 3A). Phylogenetic tree analysis revealed that VvCYP76F14s derived from 54 wine grape varieties or superior lines were categorized as neutral, aromatic, and full-bodied types (Figure 3B). Notably, the neutral- and aromatic-type berries were further divided into I, II, and III subclassifications, respectively, and the amino acid sequences of VvCYP76F14s from each subclassification were found to be identical (Supporting Information Figure S1).

In comparison to “L35” (full-bodied type), 17 (N46S, T107I, N111K, I120L, R130I, R175Q, L222V, M264I, S286N, L298 V, K325T, L371S, E378G T380A, E383D, T386A, and A437P), 14 (N46S, T107I, N111K, I120L, R175Q, L222V, M264I, S286N, L298 V, K325T, E378G, T380A, E383D, and T386A), and 15 (N46S, T107I, N111K, I120L, S140P, R175Q, L222V, Q230R, G235S, M264N, K325T, E378G, T380A, E383D, and T386A) amino acid substitutions were identified in VvCYP76F14 proteins derived from neutral I, II, and III berries, respectively. In addition, 12 (N46S, T107I, N111K, R175Q, L222V, M264I, S286N, K325T, E378G, T380A, E383D, and T386A), 16 (N46S, T107I, N111K, N134D, R175Q, T188L, L194F, L222V, M264I, S286N, K325T, E378G, T380A, E383D, T386A, and N411I), and 15 (N46S, T107I, N111K, L222V, R175Q, L194F, M264I, S286N, K325T, V368S, E378G, T380A, E383D, T386A, and F435L) amino acid substitutions were observed in VvCYP76F14 proteins derived from aromatic I, II, and III berries, respectively (Figure 3A,B). Furthermore, the expression levels of VvCYP76F14 in all these varieties or superior lines showed no significant differences in the tested berries (Supporting Information Figure S2).

### Distinct Key Amino Acid Substitutions in VvCYP76F14 Responsible for the Decreased Levels of Wine Lactone Precursors in Neutral- and Aromatic-Type Berries

**Figure 4:**
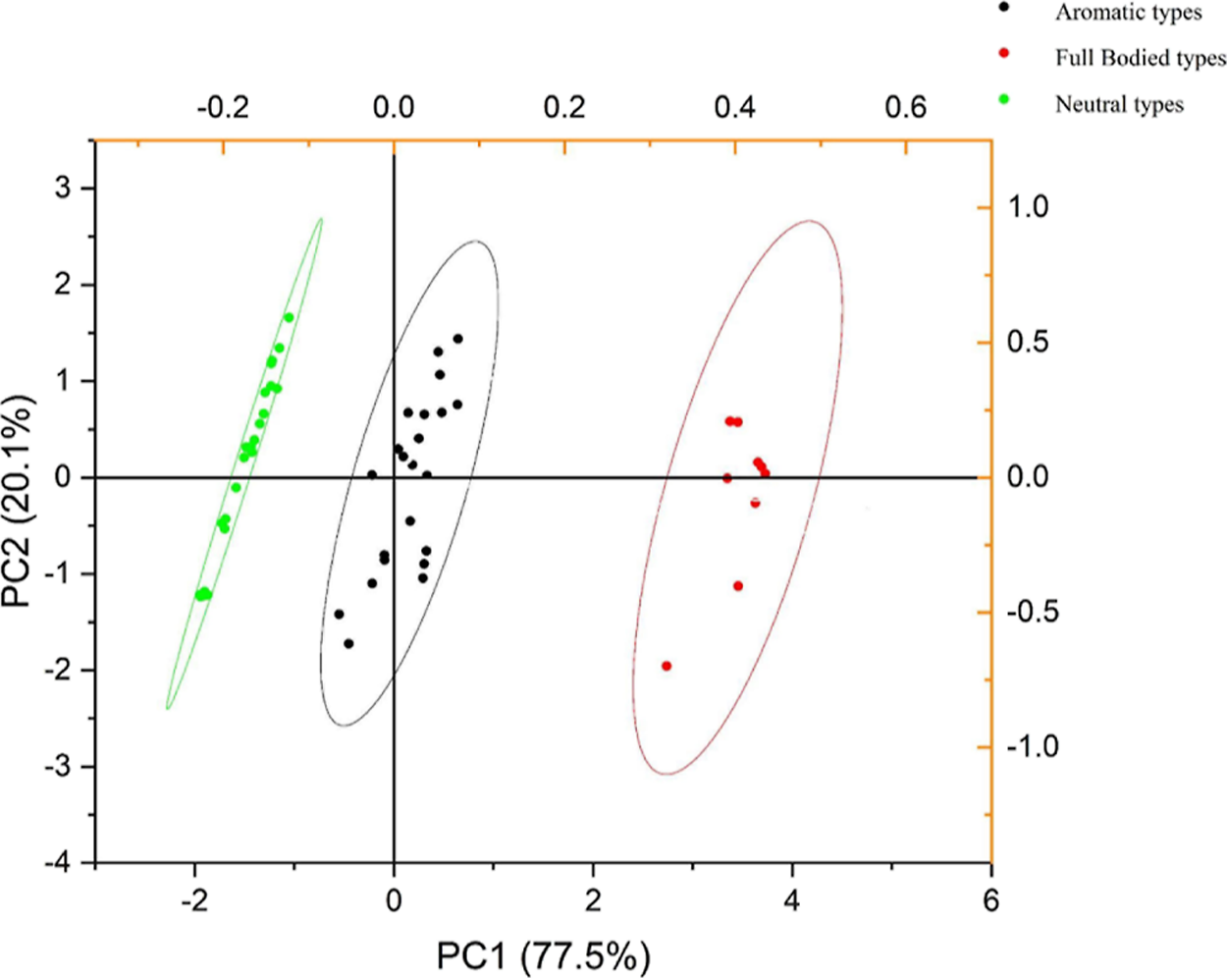
PCA of 54 wine grape varieties based on wine lactone precursors of (*E*)-8-hydroxylinalool, (*E*)-8-oxolinalool, and (*E*)-8-carboxylinalool.

A PCA using Euclidean similarity indices of (*E*)-8-hydroxylinalool, (*E*)-8-oxolinalool, and (*E*)-8-carboxylinalool wine lactone precursors successfully classified these 54 varieties or superior lines into three distinct groups, completely consistent with the categories of neutral, aromatic, and full-bodied types (Figure 4). Importantly, these three groups corresponded to the sensory evaluation of wine bouquet intensity, and distinct key amino acid substitutions in VvCYP76F14 are responsible for the decreased levels of wine lactone precursors in neutral- and aromatic-type berries (Table 1), implying a strong correlation between the substitutions of key amino acid residues in VvCYP76F14s derived from different varieties or superior lines and the intensity of wine bouquet (Figure 3 and Table 1).

### Computer Modeling, Molecular Docking, and Molecular Dynamics Simulation Analysis Revealing Key Amino Acid Residues Involved in Each Reaction of VvCYP76F14

To determine the predicted catalytic mechanism of the full-bodied type VvCYP76F14 and identify which amino acids participate in substrate–enzyme recognition, analyses of computer modeling, molecular docking, and molecular dynamics simulations were performed based on the amino acid sequence of “L35” VvCYP76F14 (full-bodied). The stereochemical quality analysis showed that 98.60% of the amino acid residues of the “L35” VvCYP76F14 model’s structure were within the reasonable range. Verification of the “L35” VvCYP76F14 modeling analysis showed that 89.70% of the amino acid residues scored above 0.2, which met the evaluation requirements. Therefore, the 3D structure of “L35” VvCYP76F14 was accepted for subsequent analyses.

**Figure 5:**
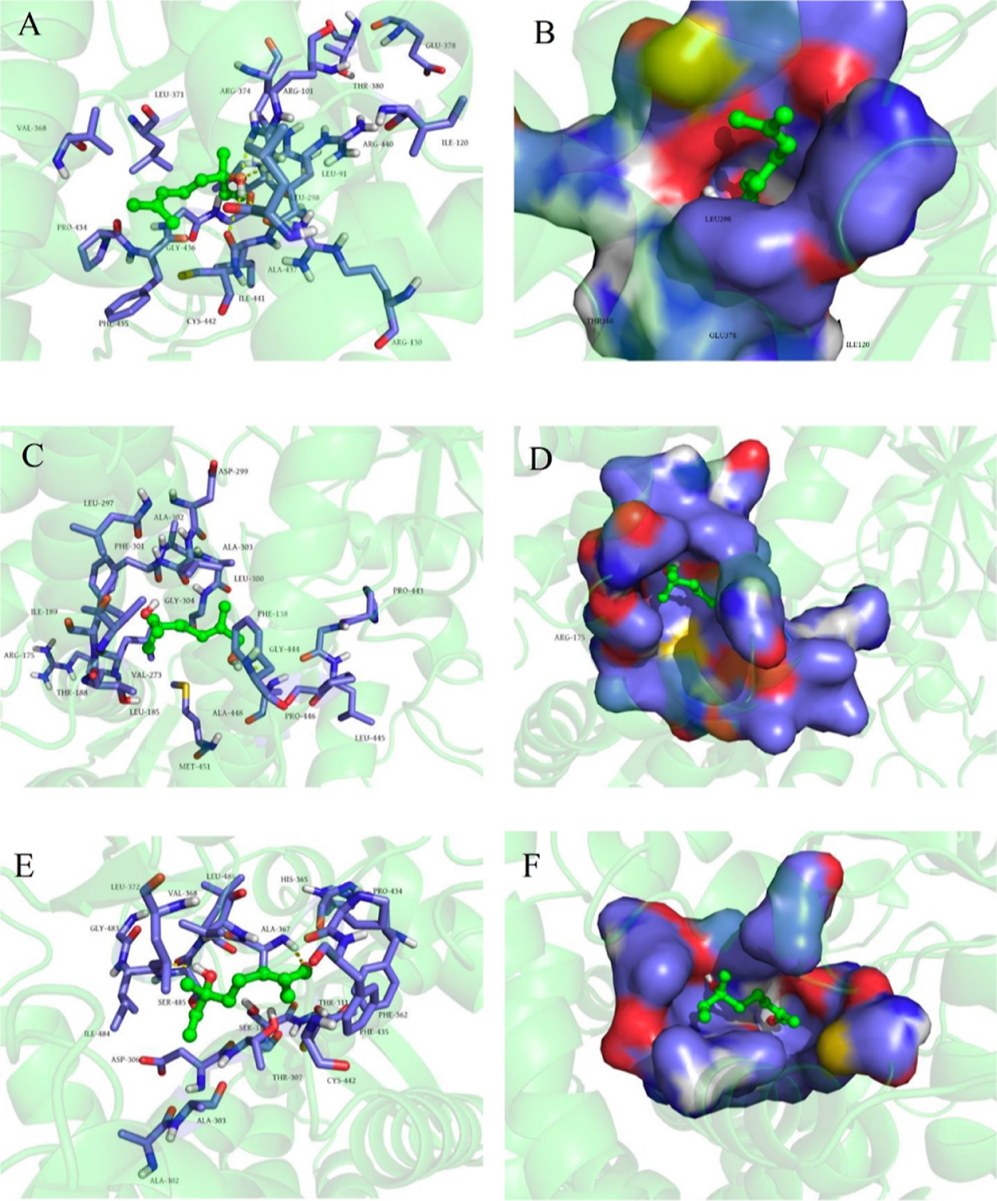
Computer modeling and molecular docking-based structural analysis revealed key amino acid residues involved in the monooxygenase reaction of VvCYP76F14. (A,B) Prediction of amino acid residues involved in the catalytic process during substrate binding to the active site of VvCYP76F14. (C,D) Prediction of amino acid residues involved in the CYP76F14–(*E*)-8-hydroxylinalool complexes. (E,F) Prediction of amino acid residues involved in the biosynthesis of (*E*)-8-carboxylinalool using (*E*)-8-oxolinalool as the substrate.

In the VvCYP76F14–linalool complexes, 17 amino acid residues were predicted to be involved in catalysis during the binding of the substrate to the VvCYP76F14 active site (Figure 5A). Compared to the amino acid sequence of the representative full-bodied “L35” VvCYP76F14, 4 substitutions (I120L, L298V, E378G, and T380A) were observed in VvCYP76F14 from neutral berries (“Yantai 1-2-13”, “Cabernet Franc”, and “Yantai 2-3-37”) and 2 substitutions (E378G and T380A) were found in VvCYP76F14 from aromatic berries (“Pinot Gris”, “Yantai 1-2-8”, and “Yantai 2-2-2”) (Figure 5B). For the subsequent dehydrogenation reaction, 19 amino acid residues were obtained in the VvCYP76F14–(*E*)-8-hydroxylinalool complexes, with a substitution (R175Q) observed in VvCYP76F14 from both neutral and aromatic types, compared to the representative full-bodied “L35” VvCYP76F14 (Figure 5C,D). Finally, 18 amino acid residues were predicted to be implicated in the biosynthesis of (*E*)-8-carboxylinalool using (*E*)-8-oxolinalool as the substrate (Figure 5E,F). Interestingly, 4 residues (V368, P434, F435, and C442) were predicted to be involved in both VvCYP76F14–linalool and VvCYP76F14–(*E*)-8-oxolinalool reactions, and 2 residues (A302 and A303) were found to participate in both VvCYP76F14–(*E*)-8-hydroxylinalool and VvCYP76F14–(*E*)-8-oxolinalool reactions (Figure 5).

A molecular mechanics–generalized Born surface area (MM–GBSA) analysis was conducted to estimate the relative binding free energy (Δ*G*_bind_) of the VvCYP76F14–ligand complexes. For each VvCYP76F14–ligand reaction system, the contributions of van der Waals (vdW), electrostatic interactions, and solvation (polar and nonpolar) were calculated to gain insights into the molecular conformations (Table 2). Among these, the van der Waals (Δ*H*_MM_^vdW^) component made the most significant contribution to the total free energy of each complex. In the dehydrogenation reaction system, the interaction energy of the VvCYP76F14–(*E*)-8-hydroxylinalool complex was found to be stable (−84.11 ± 1.66 kcal/mol).

**Table 2:** MM–GBSA Analysis of VvCYP76F14–Ligand Complexes (kcal/mol)^a^

complexes	Δ*H*_MM_^vdW^	Δ*G*_sol-pol_	Δ*G*_sol-npol_	Δ*H*_MM_^ele^	Δ*G*_bind_
VvCYP76F14–linalool	–54.59 ± 4.29	23.25 ± 1.71	–16.22 ± 0.11	–33.41 ± 2.55	–80.83 ± 3.89
VvCYP76F14–(*E*)-8-hydroxylinalool	–46.35 ± 1.97	19.22 ± 1.01	–14.59 ± 0.09	–42.18 ± 2.99	–84.11 ± 1.66
VvCYP76F14–(*E*)-8-oxolinalool	–41.26 ± 1.52	16.78 ± 1.21	–14.13 ± 0.06	–38.62 ± 1.26	–78.51 ± 1.64

a Δ*H*_MM_^vdW^, Δ*H*_MM_^ele^, Δ*G*_sol-pol_, Δ*G*_sol-npol_, and Δ*G*_bind_ represent van der Waals contribution, electrostatic contribution, polar solvation, nonpolar solvation, and free energy of substrate binding in the protein–ligand complex.

### Substitution of Key Amino Acid Residues Decreases VvCYP76F14’s Enzymatic Activity In Vitro

To estimate the individual contribution of each candidate amino acid residue to the reactions of “L35” VvCYP76F14, their functions were examined using site-directed mutagenesis. VvCYP76F14 and VvCYP76F14-SMs were expressed in *E. coli* as above and in vitro enzyme activity levels of recombinant VvCYP76F14 from “L35” (full-bodied) and VvCYP76F14-SMs were further independently assayed using linalool, (*E*)-8-hydroxylinalool, and (*E*)-8-oxolinalool as the substrate, respectively.

According to the enzyme kinetic analyses, the independent substitution of V368, L371, and P434 did not produce significant effects on the affinity (*k*_m_ values), turnover number (*k*_cat_), or enzyme efficiency (*k*_cat_/*k*_m_ ratios) in the hydroxylation reaction in which linalool was the substrate (Table 3). However, independent substitutions of the other 14 candidate amino acid residues resulted in a significant decrease in the *k*_cat_/*k*_m_ ratios, indicating reduced enzymatic activities in the corresponding VvCYP76F14-SMs. Notably, independent substitutions of all 19 candidate amino acid residues, except A302, L445, and A448, were found to decrease the *k*_cat_/*k*_m_ ratio when (*E*)-8-hydroxylinalool was used as the substrate. In particular, the D299 substitution resulted in a complete loss of enzymatic activity. In the VvCYP76F14-(*E*)-8-oxolinalool carboxylation reaction, independent substitution of each candidate amino acid residue, with the exception of I484, led to decreased enzymatic activity in the corresponding VvCYP76F14-SMs (Table 3).

**Table 3:** Enzyme Kinetics of VvCYP76F14 and Its Site-Directed Mutant Proteins (VvCYP76F14-SMs) Using Linalool, (*E*)-8-Hydroxylinalool, and (*E*)-8-Oxolinalool as the Substrate, Respectively^a^

complexes	enzyme	*k*_m_ (μM)	*k*_cat_ (S^–1^)	*k*_cat_/*k*_m_
VvCYP76F14–linalool	VvCYP76F14	66.2416 ± 6.6288^c^	13.1259 ± 1.5914^a^	0.2173 ± 0.0182^a^
	L91A	136.2555 ± 17.5441^b^	10.2917 ± 1.0552^ab^	0.0795 ± 0.0081^b^
	R101A	112.1955 ± 9.2164^bc^	5.5572 ± 0.6169^bc^	0.0478 ± 0.0036^b^
	I120L	156.2988 ± 12.7668^b^	6.2449 ± 0.6668^bc^	0.0433 ± 0.0037^b^
	R130A	70.5513 ± 6.2680^c^	6.7952 ± 0.4782^b^	0.0965 ± 0.0082^b^
	L298V	74.6625 ± 7.8927^c^	0.1982 ± 0.0155^c^	0.0013 ± 0.0002^c^
	V368S	69.2416 ± 5.2617^c^	12.9366 ± 1.4221^a^	0.1903 ± 0.0216^a^
	L371A	72.9266 ± 5.4492^c^	12.7961 ± 0.9223^a^	0.1799 ± 0.0105^a^
	R374A	105.3278 ± 8.5529^bc^	4.3889 ± 0.2677^bc^	0.0418 ± 0.0032^b^
	E378G	124.2018 ± 9.5996^bc^	1.1022 ± 0.1031^c^	0.0088 ± 0.0006^c^
	T380A	163.3218 ± 12.2116^b^	7.1588 ± 0.6524^b^	0.0439 ± 0.0041^b^
	P434V	71.5500 ± 6.1309^c^	11.1547 ± 0.8211^a^	0.1549 ± 0.0173^a^
	F435A	288.44 ± 19.3296^a^	9.7516 ± 0.8499^ab^	0.0331 ± 0.0024^b^
	G436A	194.1433 ± 13.1422^a^	7.9412 ± 0.655^b^	0.0419 ± 0.0036^b^
	A437S	118.2164 ± 8.9522^bc^	5.3106 ± 0.4397^bc^	0.0493 ± 0.0039^b^
	R440V	193.0169 ± 12.7365^ab^	1.3730 ± 0.0938^c^	0.0073 ± 0.0006^c^
	I441V	153.2964 ± 9.7523^b^	6.7752 ± 0.1987^b^	0.0451 ± 0.0062^b^
	C442A	129.1577 ± 7.6672^bc^	6.6573 ± 0.6338^b^	0.0522 ± 0.0037^b^
VvCYP76F14–(*E*)-8-hydroxylinalool	VvCYP76F14	29.1227 ± 1.6672^c^	13.5572 ± 0.6672^a^	0.4713 ± 0.0228^a^
	F138A	101.1447 ± 9.2287^b^	7.3112 ± 0.6008^b^	0.0741 ± 0.0059^b^
	R175Q	178.4463 ± 18.5224^a^	2.3112 ± 0.1822^c^	0.0127 ± 0.0011^c^
	L185A	69.5833 ± 5.1458^bc^	6.9224 ± 0.7582^b^	0.1113 ± 0.0129^b^
	T188A	77.3618 ± 8.5589^bc^	3.2282 ± 0.4553^c^	0.0424 ± 0.0061^b^
	I189A	116.5262 ± 8.2260^ab^	10.1131 ± 1.1229^ab^	0.0087 ± 0.0011^c^
	V273A	44.8556 ± 1.2669^c^	1.4421 ± 0.08554^c^	0.0321 ± 0.0024^b^
	L297A	93.1443 ± 7.2388^b^	9.5598 ± 0.6991^ab^	0.1029 ± 0.0094^b^
	D299A	N.D	N.D	N.D
	L300A	102.4223 ± 7.6328^b^	10.5237 ± 1.2574^a^	0.1077 ± 0.0086^b^
	F301A	87.5527 ± 5.1062^b^	3.1156 ± 0.2247^c^	0.0355 ± 0.0034^b^
	A302S	37.5583 ± 3.9952^c^	11.2788 ± 1.4483^ab^	0.3001 ± 0.0291^a^
	L303A	31.1478 ± 1.3648^c^	1.5591 ± 0.1338^c^	0.0511 ± 0.0037^b^
	G304A	169.7758 ± 13.5527^b^	3.1128 ± 0.4882^c^	0.0183 ± 0.0016^b^
	P443A	133.2279 ± 10.4558^ab^	6.7114 ± 0.8113^b^	0.0503 ± 0.0069^b^
	G444A	96.4488 ± 10.1227^b^	5.339 ± 0.4881^bc^	0.0553 ± 0.0049^b^
	L445A	33.9633 ± 1.7458^c^	10.1722 ± 1.1068^ab^	0.2994 ± 0.0201^a^
	P446A	117.3679 ± 8.5547^ab^	6.2274 ± 0.5877^b^	0.0534 ± 0.0038^b^
	A448S	39.7761 ± 1.7458^c^	11.2544 ± 0.8662^a^	0.2829 ± 0.0213^a^
	M451A	70.2231 ± 6.8827^bc^	4.4432 ± 0.4062^bc^	0.0632 ± 0.0047^b^
VvCYP76F14–(*E*)-8-oxolinalool	VvCYP76F14	41.4482 ± 4.9338^c^	11.1129 ± 1.0221^a^	0.2981 ± 0.0319^a^
	A302S	123.0021 ± 10.2236^bc^	7.5226 ± 0.5966^ab^	0.0720 ± 0.0052^b^
	L303A	102.5361 ± 9.8843^bc^	1.3681 ± 0.1124^c^	0.0133 ± 0.0012^c^
	D306A	269.2241 ± 17.6692^a^	0.6554 ± 0.0622^c^	0.0022 ± 0.0002^c^
	T307A	97.3619 ± 8.7298^c^	2.0018 ± 0.1446^c^	0.0236 ± 0.0019^b^
	T311A	109.2284 ± 7.7298^bc^	7.2514 ± 0.5582^ab^	0.0601 ± 0.0042^b^
	S319A	126.4111 ± 11.7554^bc^	3.2249 ± 0.2916^c^	0.0253 ± 0.0031^b^
	F362A	110.5584 ± 8.2260^bc^	5.5589 ± 0.6448^b^	0.0488 ± 0.0053^b^
	H365A	66.1812 ± 5.5547^c^	1.1156 ± 0.1019^c^	0.0169 ± 0.0020^bc^
	A367S	88.2114 ± 6.2289^c^	3.6652 ± 0.4012^bc^	0.0399 ± 0.0041^b^
	V368A	144.5594 ± 9.2246^b^	4.3226 ± 0.4472^bc^	0.0274 ± 0.0031^b^
	L372A	127.1912 ± 9.2272^b^	7.009 ± 0.5001^ab^	0.0593 ± 0.0041^b^
	P434V	78.6684 ± 5.1302^c^	7.0521 ± 0.8118^ab^	0.0891 ± 0.0122^b^
	F435A	133.2741 ± 10.1006^b^	5.2169 ± 0.4892^b^	0.0397 ± 0.0028^b^
	C442A	206.1154 ± 17.2234^a^	3.6338 ± 0.2118^b^	0.0184 ± 0.0012^bc^
	G483A	141.5224 ± 9.1425^b^	8.1023 ± 0.7116^a^	0.0572 ± 0.0050^b^
	I484A	56.3324 ± 3.4752^c^	10.6698 ± 1.2289^a^	0.2109 ± 0.0198^a^
	S485A	171.5231 ± 9.9853^b^	5.3362 ± 0.6698^b^	0.0311 ± 0.0030^b^
	L486A	108.1887 ± 9.5547^bc^	6.2136 ± 0.5221^b^	0.0573 ± 0.0059^b^

a VvCYP76F14 was isolated from the full-bodied variety “L35”. Data were presented as the mean ± SE (*n* = 3). Letters indicate significant differences at a significance level of *P* ≤ 0.05, as determined using ANOVA followed by Fisher’s LSD test. N.D., not detectable.

In particular, 5 amino acid substitutions (I120L, R175Q, L298V, E378G, and T380A) observed in VvCYP76F14 from neutral and aromatic varieties were identified as key candidate amino acid residues (Figures 3 and 5,B,D). To further evaluate the impact of the remaining 10 amino acid substitutions (N46S, T107I, N111 K, R175Q, L222V, M264I, S286N, K325T, E383D, and T386A) in these neutral and aromatic varieties on VvCYP76F14’s enzymatic activity, site-directed mutagenesis experiments were conducted. However, no significant differences in the enzymatic activity were observed between the mutated variants (VvCYP76F14-SMs) and wild-type VvCYP76F14 (Supporting Information Table S1).

### D299 Mutation of VvCYP76F14 Led to the Complete Loss of (*E*)-8-Oxolinalool and (*E*)-8-Carboxylinalool Biosynthesis Activities In Vitro

Notably, site-directed mutagenesis of D299 in VvCYP76F14 led to the complete loss of (*E*)-8-oxolinalool biosynthesis activity in vitro (Table 3), aligning with the results observed in *V. vinifera* cv. “Yantai 2-3-37” (harboring the D299 mutation in VvCYP76F14) where undetectable levels of (*E*)-8-oxolinalool and (*E*)-8-carboxylinalool were produced (Table 1 and Figure 3).

## Discussion

In wine grapes, the enzyme VvCYP76F14 is implicated in three reaction processes using linalool as a substrate (hydroxylation, dehydrogenation, and oxidation).^13,15^ However, how specific amino acid residues of VvCYP76F14 contribute to the wine lactone precursor formation remains largely unknown.

Phenotypic variability in aromatic component contents due to substitutions in key enzymes has been observed in different fruit crop cultivars. In peach (*Prunus persica* L.), amino acid substitutions in alcohol acyltransferase PpAAT1 are responsible for low levels of the key aroma compound γ-decalactone in low-aroma cultivars.^38,42^ Similarly, different cultivars of apple (*Malus domestica* Borkh.) exhibit variations in the enzymatic activity of MdAAT1, which is involved in ester biosynthesis, due to AAT1 site mutations.^43^ In this study, distinct amino acid substitutions in VvCYP76F14 were identified in neutral- and aromatic-type berries, and these substitutions are responsible for decreased levels of wine lactone precursors. Mutating key enzymes to select cultivars with desired characteristics has been well documented in various crops.^38,42,44,45^ For instance, mutating glucose–methanol–choline oxidoreductase is a cost-effective method for selecting thermosensitive genic male-sterile rice cultivars,^44^ and targeted mutagenesis of the seed fatty acid reducer has been useful for improving oil yield in oil crops.^45^

Ilc et al. observed amino acid differences between two VvCYP76F14 proteins but found no significant differences in VvCYP76F14 activity in vitro.^13^ This lack of significant differences might be attributed to the insufficient amounts of recombinant enzymes required for further quantitative assays without the assistance of an MBP fusion tag. In our study, we introduced the MBP fusion tag and investigated the key amino acid residues of VvCYP76F14 obtained from a full-bodied variety, “L35”, elucidating their contribution to enzyme activity through in vitro enzymatic assays. In vitro enzymatic assays are commonly employed to characterize members of the CYP450 family in plants, including those from *A. thaliana*,^17^*Solanum miltiorrhiza*,^37^ and *Solanum tuberosum*.^46^ Furthermore, most of the substitutions of putative key amino acid residues involved in all three reactions significantly decreased the enzymatic activities of VvCYP76F14-SMs in vitro. The reactions catalyzed by VvCYP76F14 provided valuable information, suggesting its potential application as a selective marker to screen VvCYP76F14 variants in grape varieties that contribute differently to wine bouquet. However, it is important to note that the number of verified wine grape varieties is not extensive, especially for neutral-type III and aromatic-type II and III varieties. The effectiveness is limited to the 54 selected varieties or superior lines, and therefore, it may not be suitable for all wine grape varieties in nature.

Additionally, we identified differences in the amino acid sequences of VvCYP76F14s from 54 distinct varieties, comprising 24 neutral, 21 aromatic, and 9 full-bodied types. Various amino acid substitutions were found in both neutral and aromatic varieties, suggesting a close relationship among key amino acid substitutions, VvCYP76F14 activity, and linalool-derivative production. Notably, the presence of D299 in the “Yantai 2-3-37” variety and the D299T mutation resulted in undetectable levels of (*E*)-8-oxolinalool and (*E*)-8-carboxylinalool in the berries. This suggests that substitutions in VvCYP76F14 led to the loss of linalool derivatives in certain neutral varieties, highlighting the indispensable role of this amino acid residue in the biosynthesis of the wine lactone precursor. Considering that natural multisubstrate enzymes have evolved to facilitate the rate-limiting step in the enzymatic cycle, and that the product of the previous reaction may serve as the substrate for the next reaction,^1,13,18^ we speculate that linalool may bind to its specific binding site and is hydroxylated to (*E*)-8-hydroxylinalool by the VvCYP76F14-ATR1 (CPR) system. The substrate–product exchange may lead to the separation of VvCYP76F14 from ATR1 (CPR). We propose that the CYP76F14-(*E*)-8-hydroxylinalool reaction is likely to be the rate-limiting step in the overall catalytic reaction, but this hypothesis needs further verification.

Nonetheless, the identification of key amino acid substitutions in VvCYP76F14 provides an opportunity to use this enzyme as a fingerprint marker for screening hybrid offspring with desired levels of linalool derivatives.

## Data Availability

All data that support the findings of this study are available from the corresponding author upon reasonable request.

## Supporting Information Available

Enzyme kinetics of VvCYP76F14 and its site-directed mutant proteins (VvCYP76F14-SMs) using linalool, (*E*)-8-hydroxylinalool, and (*E*)-8-oxolinalool as substrates, respectively; amino acid sequence alignment of VvCYP76F14s from 54 wine grape varieties or superior lines; multiple sequence alignment performed using the ClustalW program within the MEGA 13.0 software, based on the amino acid sequences of VvCYP76F14s from 54 wine grape varieties or superior; and expression levels of VvCYP76F14s from 54 wine grape varieties or superior lines (PDF)

## Abbreviations

CDS: coding sequences
CPR: cytochrome P450 reductase
MBP: maltose-binding protein
MM–GBSA: molecular mechanics–generalized Born surface area
PCA: principal coordinate analysis
qPCR: real-time quantitative PCR
SM: site-directed mutagenesis
UPLC–MS: ultraperformance liquid chromatography–mass spectrometry
