# Supplementary material for: Site-Directed Mutagenesis of VvCYP76F14 (Cytochrome P450) Unveils Its Potential for Selection in Wine Grape Varieties Linked to the Development of Wine Bouquet
Source: J Agric Food Chem. 2024 Feb 9;72(7):3683–94. doi: 10.1021/acs.jafc.3c09083 (PMC10885137; doi:10.1021/acs.jafc.3c09083)
Supplement: Supplementary file 1 — jf3c09083_si_001.pdf [file jf3c09083_si_001.pdf]

Supporting information

Site-directed mutagenesis of VvCYP76F14 (cytochrome P450) unveils its potential for selection in wine grape varieties linked to the development of wine bouquet

Bin Peng, Jianguo Ran, Yiyang Li, Meiling Tang, Huilin Xiao, Shengpeng Shi, Youzheng Ning, Adeeba Dark, Jin Li, Xueqiang Guan, Zhizhong Song*

The Engineering Research Institute of Agriculture and Forestry, Ludong University, Yantai 264025, China

Cocodala Vocational and Technical College, Cocodala 853213, China

Jiangsu Vocational College of Agriculture and Forestry, Zhenjiang 212499, China

Yantai Academy of Agricultural Sciences, Yantai 265599, China

Department of Plant Science, University of Cambridge, Cambridge CB2 3EA, UK

Shandong Technology Innovation Center of Wine Grape and Wine/ COFCO Great Wall Wine (Penglai) Co., Ltd, Yantai 264000, China

***Correspondence:**

zs316@cam.ac.uk.

Supplementary Table S1. Enzyme kinetics of VvCYP76F14 and its site-directed mutant proteins (VvCYP76F14-SMs) using linalool, (*E*)-8-hydroxylinalool, and (*E*)-8-oxolinalool as substrate, respectively.

Complexes	Enzyme	k_m (μM)	k_{cat} (S^{-1})	k_{cat}/k_m
VvCYP76F14-linalool	VvCYP76F14	66.2416 ± 5.6288[a]	13.1259 ± 1.5914[a]	0.2173 ± 0.0172[a]
	N46S	67.2188 ± 7.1121[a]	13.6507 ± 1.8543[a]	0.2017 ± 0.0214[a]
	T107I	68.1873 ± 6.4128[a]	12.1824 ± 0.9672[a]	0.1816 ± 0.0139[a]
	N111K	69.3129 ± 8.2219[a]	12.5536 ± 1.2217[a]	0.1792 ± 0.0152[a]
	R175Q	70.4162 ± 6.7246[a]	10.7644 ± 0.9668[a]	0.1852 ± 0.0138[a]
	L222V	67.5229 ± 7.1168[a]	13.5129 ± 1.1125[a]	0.2019 ± 0.0183[a]
	S286N	71.2542 ± 8.2210[a]	12.2295 ± 0.9100[a]	0.1731 ± 0.0129[a]
	K325T	67.2892 ± 6.5331[a]	11.1552 ± 0.8644[a]	0.1669 ± 0.0118[a]
	E383D	72.3824 ± 6.9140[a]	13.0045 ± 1.1126[a]	0.1712 ± 0.0151[a]
	T386A	65.1396 ± 7.2251[a]	12.3144 ± 1.1054[a]	0.1855 ± 0.0131[a]

VvCYP76F14-(*E*)-8-hydroxylinalool	VvCYP76F14	29.1227 ± 2.6672[a]	13.5572 ± 1.1672[a]	0.4713 ± 0.0228[a]
	N46S	31.3661 ± 1.1442[a]	12.5512 ± 0.8552[a]	0.4105 ± 0.0275[a]
	T107I	30.1662 ± 1.6292[a]	11.4417 ± 1.1175[a]	0.3833 ± 0.0162[a]
	N111K	29.3662 ± 2.8148[a]	12.4958 ± 1.0080[a]	0.4261 ± 0.0283[a]
	R175Q	29.5842 ± 2.5514[a]	13.6614 ± 1.6571[a]	0.4419 ± 0.0391[a]
	L222V	30.8442 ± 1.0813[a]	11.0071 ± 1.4421[a]	0.3907 ± 0.0304[a]
	S286N	29.9804 ± 2.7133[a]	13.8802 ± 1.2017[a]	0.4385 ± 0.0201[a]
	K325T	31.2268 ± 3.7746[a]	12.5533 ± 1.1164[a]	0.4019 ± 0.0348[a]
	E383D	30.1509 ± 2.8865[a]	13.0119 ± 1.0040[a]	0.4308 ± 0.0188[a]
	T386A	31.9552 ± 3.5861[a]	11.9365 ± 1.1466[a]	0.3779 ± 0.0401[a]
VvCYP76F14-(*E*)-8-oxolinalool	VvCYP76F14	41.4482 ± 2.9338[a]	11.1129 ± 1.0221[a]	0.2981 ± 0.0239[a]
	N46S	45.3384 ± 5.1007[a]	10.1887 ± 1.2754[a]	0.2239 ± 0.0388[a]
	T107I	42.1008 ± 4.1446[a]	9.2541 ± 1.1026[a]	0.2209 ± 0.0310[a]

N111K	41.9422 ± 3.0319[a]	11.2541 ± 1.6688[a]	0.2701 ± 0.0108[a]
R175Q	40.4128 ± 2.9552[a]	10.3321 ± 1.072[a]	0.2556 ± 0.0104[a]
L222V	42.4918 ± 3.4452[a]	10.1429 ± 1.5521[a]	0.2363 ± 0.0185[a]
S286N	43.8816 ± 3.5009[a]	13.1842 ± 1.6687[a]	0.3001 ± 0.0284[a]
K325T	41.6675 ± 2.7114[a]	12.2219 ± 1.0317[a]	0.2914 ± 0.0192[a]
E383D	43.1147 ± 4.2217[a]	11.9962 ± 0.9987[a]	0.2801 ± 0.0215[a]
T386A	41.7668 ± 3.0509[a]	10.1144 ± 0.8552[a]	0.2431 ± 0.0268[a]

VvCYP76F14 was isolated from the Full-Bodied variety ‘L35’. Data were presented as the means ± SE (*n* = 3). Letters indicate significant differences at a significance level of $P \leq 0.05$, as determined using ANOVA followed by Fisher’s LSD test.

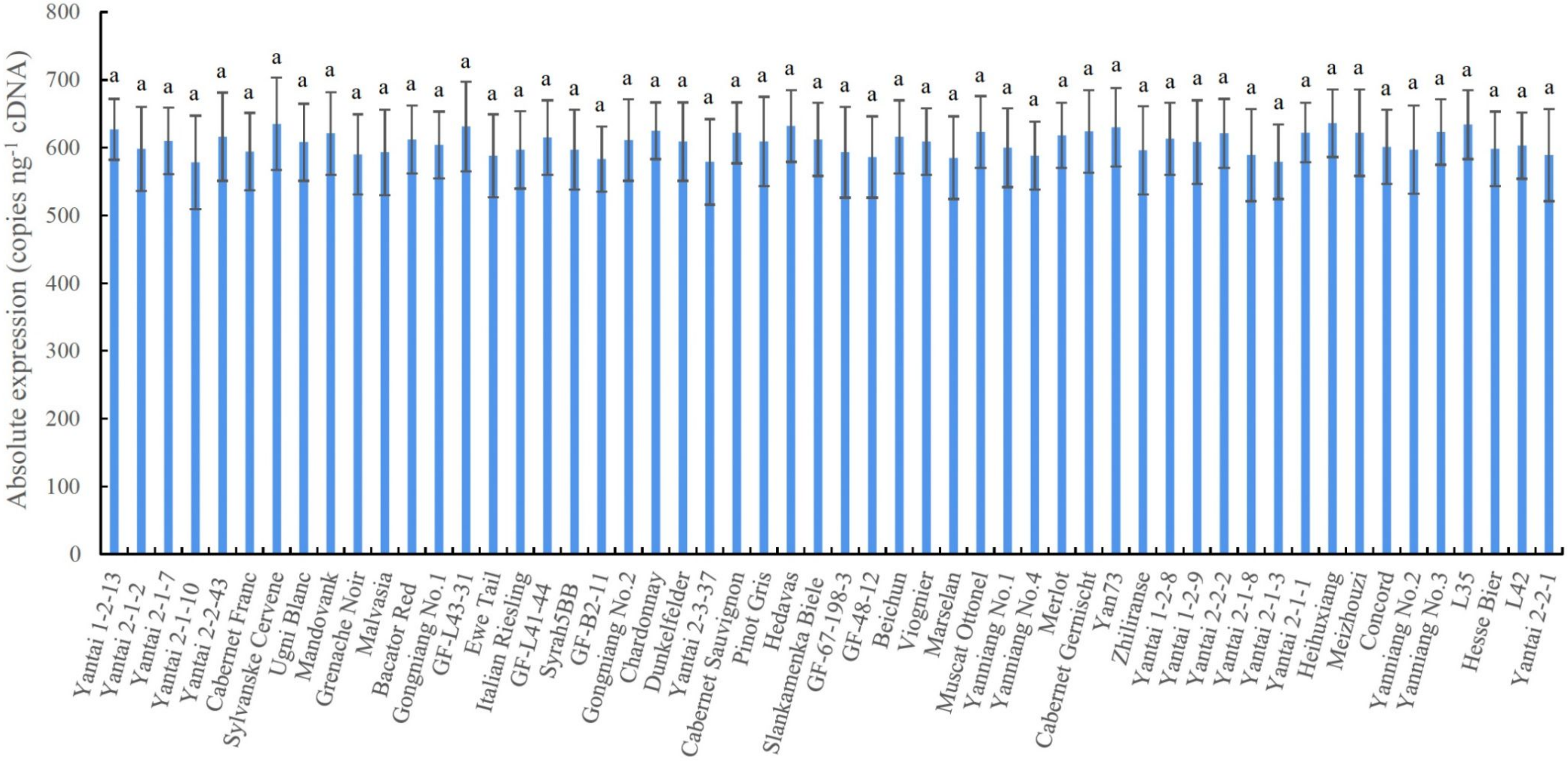

Supplementary Figure S1

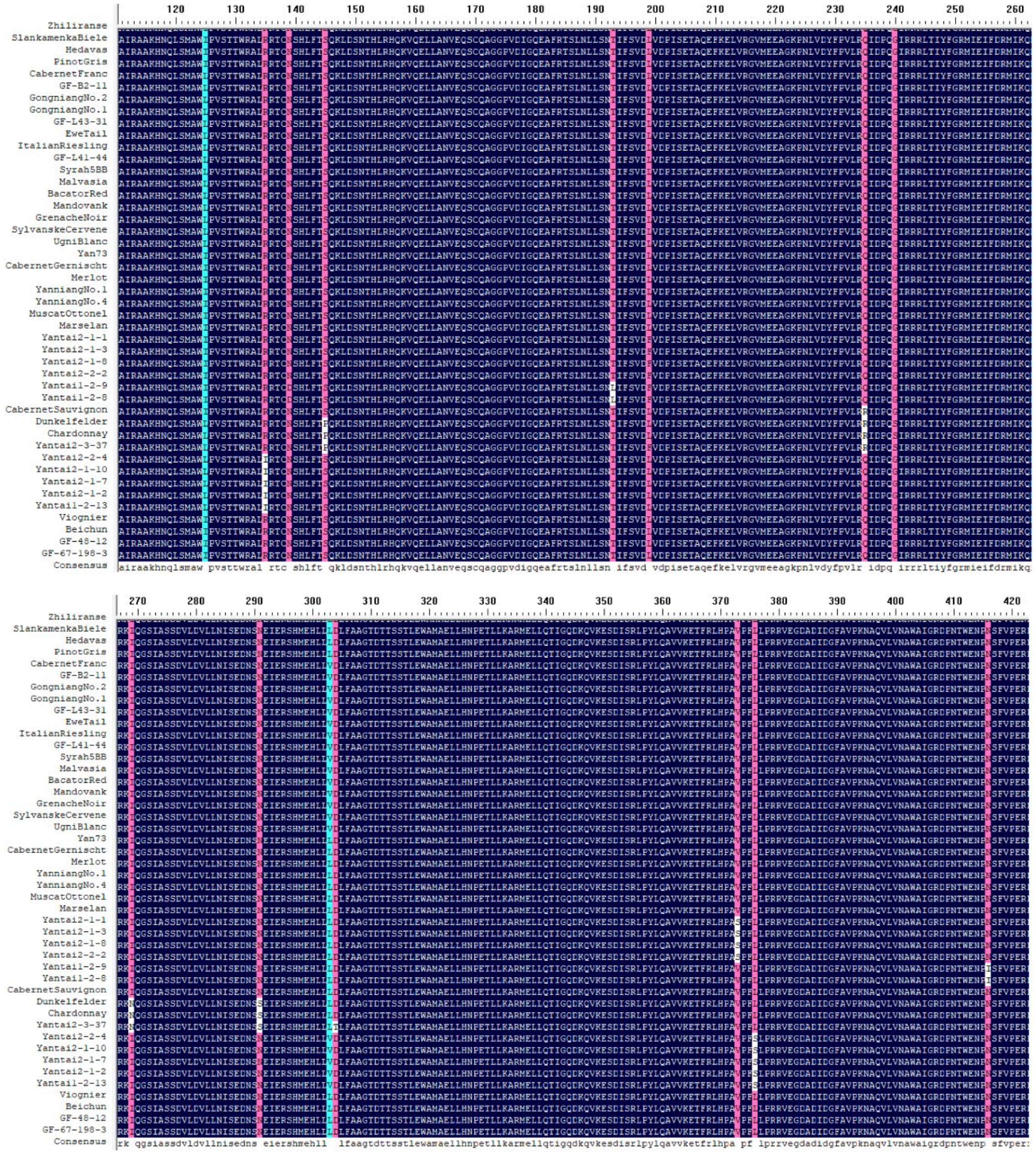
Zhiliranse
SlankamenkaBiele
Hedavas
PinotGris
CabernetFranc
GF-B2-11
GongniangNo.2
GongniangNo.1
GF-L43-31
EweTail
ItalianRiesling
GF-L41-44
Syrah5BB
Malvasia
BacatorRed
Mandovank
GrenacheNoir
SylvanskeCervene
UgniBlanc
Yan73
CabernetGernischt
Merlot
YanniangNo.1
YanniangNo.4
MuscatOttonel
Marselan
Yantai2-1-1
Yantai2-1-3
Yantai2-1-8
Yantai2-2-2
Yantai1-2-9
Yantai1-2-8
CabernetSauvignon
Dunkelfelder
Chardonnay
Yantai2-3-37
Yantai2-2-4
Yantai2-1-10
Yantai2-1-7
Yantai2-1-2
Yantai1-2-13
Viognier
Beichun
GF-48-12
GF-67-198-3
Consensus
airaakhnqlsmaw pvsttwral rtc shlft qkldsnthlrhqkvqellanveqscqaggpvdigqeafrtslnllsn ifsvd vdpisetaqefkelvrgvmeeagkpnlvdyfpvlr idpq irrrltiyfgrmieifdrmikq
rk qgsiassdvldvllnisedns eiershmehll lfaagtdttsstlewamaellhnpetllkarmellqtigqdkqvkesdisrlpylqavvketfrlhpa pf lprrvegdadidgfavpknaqvlvnawaigrdpntwenp sfvper

Supplementary Figure S2
